# Profiling the Onco-metabolic Nexus and Improving Cancer Risk Prediction Performance: A Large-scale Cohort and Genome-Wide Pleiotropic Analysis

**DOI:** 10.1158/2767-9764.CRC-26-0099

**Published:** 2026-05-08

**Authors:** Xiaolong Ji, Yixing Yang, Mengxue Qiu, Shuhan Zhao, Yu Wang, Shui Wang, Xiao Li, Peng Huang, Hui Xie, Ziyi Fu

**Affiliations:** 1 Department of Epidemiology, Centre for Global Health, School of Public Health, Nanjing Medical University, Nanjing, China.; 2 The First School of Clinical Medicine, Nanjing Medical University, Nanjing, China.; 3 Department of Breast Surgery, Jiangsu Cancer Hospital, Jiangsu Institute of Cancer Research, Affiliated Cancer Hospital of Nanjing Medical University, Nanjing, China.; 4 Department of Scientific Research, Jiangsu Key Laboratory of Innovative Cancer Diagnosis and Therapeutics, Jiangsu Cancer Hospital, Jiangsu Institute of Cancer Research, The Affiliated Cancer Hospital of Nanjing Medical University, Nanjing, China.

## Abstract

Early screening and targeted intervention can effectively reduce cancer burden. However, most studies have proposed polygenic risk score (PRS) to perform risk prediction and population risk stratification. In this study, we investigate the causal links and shared genetics between cancer and metabolic traits to map the onco-metabolic nexus. Using multivariable Cox models, we assessed 240 trait–cancer associations. Genomic analyses included genome-wide and local genetic correlations and genomic structural equation modeling (gSEM) to identify pathways linking metabolic traits to cancer. We then used Deterministic Bayesian Sparse Linear Mixed Model (DBSLMM) to build both single and integrative PRS models based on gSEM and compared their predictive performance. Most of the metabolic traits are risk factors to cancer, such as waist–hip ratio–colorectal cancer [hazard ratio (HR) = 1.34; 95% confidence interval (CI) = 1.23–1.46; *P* = 1.59 × 10^−11^]. In the genetic correlation analysis, we identified 41 significant pairs in the onco-metabolic nexus and 405 significant genomic regions. Mendelian randomization analysis revealed 17 significant causal pairs. The integrative PRS model combining gSEM for metabolic traits improved prediction, with 13.95% variance explained in kidney cancer. The study highlights the intertwined genetic and clinical relationships between cancers and metabolic traits, improving cancer screening and intervention.

**Significance:**

By uncovering the shared genetic basis of cancer and metabolic traits, our work enables a novel integrative risk prediction model, which promises to enhance precision screening and targeted prevention strategies for high-risk populations.

## Introduction

Early screening and precise targeted intervention are effective strategies to reduce the cancer burden, with their clinical value continuously demonstrated across diverse tumor types (1, 2). Emerging evidence underscores that metabolic dysregulation mediates tumor pathogenesis by remodeling the local microenvironment into a tumor pathogenic niche (3–5), highlighting the potential utility of metabolic indicators for tumor risk prediction. The proliferation of genome-wide association studies (GWAS) from case–control study and large-scale cohort studies characterizes the shared genetic basis between complex diseases and provides a scientific basis for individualized risk stratification and intervention (6, 7). Shared genetic basis, a crucial aspect of comorbidity, is useful in understanding the etiology, diagnosis, and treatment of the 2 traits (8). For example, GWAShug integrated 7 popular tools to elucidate it between 539 complex traits (9). Additionally, the polygenic risk score (PRS) has been widely used for disease risk stratification, offering novel perspectives for individualized cancer risk assessment (10–12). For instance, Zhu and colleagues (13) developed population-specific pan-cancer PRSs using the China Kadoorie Biobank, demonstrating that leveraging shared genetic susceptibility across cancers significantly enhances prediction accuracy.

Traditional GWAS methods merely capture the complex interplay between diverse metabolic measures and their collective impact on cancer risk. Moreover, they struggle to depict the comprehensive landscape of how these factors interact and influence each other through biological pathways (14–16). Integrating various metabolic traits, the genomic structural equation model (gSEM) uncovers hidden patterns, or latent factor, with advanced statistical modeling to provide deeper insights into (i) how metabolic traits relate to each other; (ii) their combined effects on cancer risk; and (iii) the underlying biological mechanisms. gSEM offers more transparent insights and enhanced predictive accuracy with regard to metabolic–cancer associations. It is especially valuable in identifying shared genetic connections between metabolic disorders and cancer, paving the way for more targeted preventive strategies and personalized treatments (17, 18).

**Figure 1.**
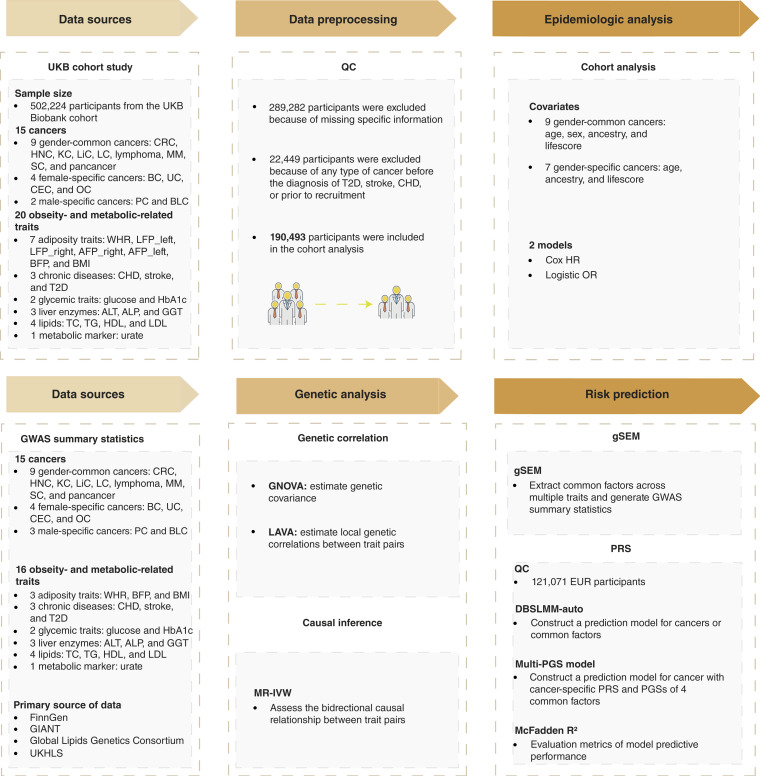
Workflow of the study. We first obtained data on cancer, metabolic traits, and genotypes from the UKB. To define the final analysis set, we applied QC procedures to exclude ineligible participants, resulting in a cohort of 190,493 individuals for subsequent epidemiologic analyses. To investigate the effects of metabolic traits on different cancers, we jointly evaluated associations using HRs from Cox regression models and ORs from logistic regression models. To explore genetic relationships between these two types of traits, we collected GWAS summary data from EUR-ancestry populations excluding UKB for each trait. We used GNOVA to assess genome-wide genetic correlations and LAVA to examine local genetic correlations at specific genomic regions. To infer causal relationships, we applied MR with IVW (MR-IVW) methods, incorporating instrumental variables. Finally, to improve cancer risk prediction by integrating multiple metabolic indicators, we employed a gSEM to extract common latent factors across metabolic traits and constructed PRSs based on these latent factors for cancer risk prediction. BC, breast cancer; BLC, bladder cancer; CHD, chronic ischemic heart disease; CEC, cervical cancer; CRC, colorectal cancer; HNC, head and neck cancer; KC, kidney cancer; LC, lung cancer; LiC, liver cancer; OC, ovarian cancer; PC, prostate cancer; SC, stomach cancer; UC, uterine cancer; UKHLS, UK Household Longitudinal Study.

Thus, we performed an integrative analysis of large-scale cohort and GWAS summary statistics to identify the onco-metabolic nexus across different types of cancer and enhance the cancer risk prediction (Fig. 1). We used both Cox proportional hazard regression for cohort data and two-sample Mendelian randomization (MR) with GWAS summary statistics to define the casual relationship between 20 metabolic traits and 12 cancer incidences. Then, we used Genetic Covariance Analyzer (GNOVA) and Local Analysis of (co)Variant Association (LAVA) to elucidate the genetic correlation among the nexus (19, 20). Finally, using gSEM, we further investigated the genetic correlation and constructed PRS for each cancer (21, 22). This metabolic-driven strategy pioneers a novel paradigm for preventing metabolism-associated cancers.

## Materials and Methods

### Study participants

The UK Biobank (UKB) is a national prospective cohort involving a sample of 502,224 participants 37 to 73 years of age who were recruited between 2006 and 2010 (application ID: 144904). Sociodemographic information, lifestyle factors, medical history, physical and functional measures, and biological samples were collected from participants at baseline or follow-up assessments across 22 assessment centers in England, Scotland, or Wales (23).

### Metabolic measurement

We selected 20 metabolic traits in UKB: body mass index (BMI; field ID: p21002_i0), total cholesterol (TC; field ID: p23400_i0), triglycerides (TG; field ID: p23407_i0), high-density lipoprotein cholesterol (HDL-C; field ID: p23406_i0), low-density lipoprotein cholesterol (LDL-C; field ID: p23405_i0), body fat percentage (BFP; field ID : p23099_i0), waist–hip ratio (WHR; field ID: p48_i0 for waist circumstance and p49_i0 for hip circumstance), glucose (field ID: p30740_i0), HbA1c (field ID: p30750_i0), urate (field ID: p30880_i0), left leg fat percentage (LFP_left; field ID: p23115_i0), right leg fat percentage (LFP_right; field ID: p23111_i0), right arm fat percentage (AFP_right; field ID: p23119_i0), left arm fat percentage (AFP_left; field ID: p23123_i0), alanine aminotransferase (ALT; field ID: p30620_i0), alkaline phosphatase (ALP; field ID: p30610_i0), γ-glutamyltransferase (GGT; field ID: p30730_i0), type II diabetes (T2D; field ID: p130709), chronic ischemic heart disease (CIHD; field ID: p131307), and stroke (field ID: p131367; Supplementary Tables S1 and S2).

### Outcome measurement

For participants who experienced any event, that is, cancer diagnosis or death, survival time was defined as the duration from enrollment to the date of event occurrence. For those without the event, survival time was calculated as the time from enrollment to October 2023 (the data update date). We defined each cancer using the International Classification of Diseases (ICD) 10 code: colorectal cancer (ICD10: C18 and C19), malignant melanoma (MM; ICD10: C43), lung cancer (ICD10: C34), lymphoma (ICD10: C81–C86), kidney cancer (ICD10: C64), breast cancer (ICD10: C50), prostate cancer (ICD10: C61), cervical cancer (ICD10: C53), uterine cancer (ICD10: C55), bladder cancer (ICD10: C67), head and neck cancer (ICD10: C00–C14), stomach cancer (ICD10: C16), liver cancer (ICD10: C22), and ovarian cancer (ICD10: C56). Specifically, uterine cancer (Ncase = 8) and cervical cancer (Ncase = 47) were excluded from the analysis because of the rare number of cases. The imbalanced ratio of cases to controls might cause the estimation bias.

In addition, we set breast cancer and ovarian cancer as female-specific cancers. We also regarded prostate cancer and bladder cancer as male-specific cancers. For 8 non–sex-specific cancers, we fitted the models adjusting for age (field ID: p34), sex (field ID: p31), ancestry (field ID: p21000_i0), and healthy lifescore to account for potential confounding factors. For the 4 sex-specific cancers, we fitted the models without sex.

### GWAS summary statistics

We collected GWAS summary statistics for 12 types of cancer and 16 metabolic phenotypes from European (EUR) individuals. Due to the unavailability of suitable GWAS summary data, we excluded 4 traits: AFP_left, AFP_right, LFP_left, and LFP_right. These data were primarily sourced from large cohorts or consortia, including 16 from FinnGen r12, 1 from GIANT, 4 from the Global Lipids Genetics Consortium, 3 from the UK Household Longitudinal Study, 1 from the CKDGen Consortium, and 3 from other studies (24–27). We perform quality control (QC) by filtering out low-quality variants of reference panels based on the parameters mentioned: SNPs with a minor allele frequency <0.01, SNPs with a Hardy–Weinberg equilibrium *P* value < 10^−7^, SNPs with >5% missing data (*Pm* > 0.05), and duplicated SNPs (22, 28, 29). The remaining SNPs were compared with those in the HapMap 3 (HM3) reference panel. After filtering, we only retained 1.2 million HM3 SNPs. We calculated the heritability for each GWAS summary statistic using PLINK (v1.90b7; ref. 30) and excluded 3 GWAS summary datasets (ALT, ALP, and GGT) with negative heritability estimates. Detailed information about these datasets is provided in Supplementary Tables S3 and S4. Using liftOver, we aligned all GWAS data to the GRCh38 reference genome assembly.

### Statistical analysis

#### Prospective association between 12 cancers and 20 metabolic traits

We applied strict QC to the UKB cohort to investigate the causal relationships between 12 cancer incidences and 20 metabolic traits (Supplementary Fig. S1). We excluded participants (i) with missing specific information and (ii) with any cancer diagnosed before the onset of T2D, stroke, CIHD, or before study enrollment. We retained 190,493 individuals as the dataset for the cohort analysis (Supplementary Table S1).

The primary outcome analysis aimed to estimate hazard ratios (HR) and their 95% confidence intervals (CI) for each metabolic trait in relation to the risk of specific cancers. We used multivariable Cox proportional hazards regression models adjusting for the covariates. This approach allowed us to account for the time-to-event nature of the outcomes while adjusting for potential confounding factors. In the secondary analysis, we examined the association between disease incidence and metabolic traits. For this analysis, individuals who did not onset the disease were defined as the reference group (coded as 0). We used multivariable logistic regression models to estimate odds ratios (OR) and their corresponding 95% CIs for each metabolic trait adjusting for the covariates. In the time-to-event analysis, we defined the primary endpoint as the occurrence of the specific cancer of interest. Competing risks (e.g., noncancer death) were treated as censoring events in the Cox proportional hazards model. Individuals who experienced noncancer death during follow-up were censored at the time of death, and their follow-up time was no longer included in the risk set for cancer incidence after that point. This complementary approach provided insights into the associations between metabolic factors and the overall likelihood of cancer development, independent of the time at which the event occurred.

#### Causal relationships between 12 cancers and 13 metabolic traits

We applied a two-sample MR approach to investigate the causal relationships between 12 cancer types and 13 obesity-related traits. In the framework of FDR using the Benjamini–Hochberg (BH) procedure, we used 4 popular methods for MR analysis: MR-Egger, weighted median (WM), inverse-variance weighted (IVW-random effect), and IVW-fixed effect (31–34). With the Instrument Strength Independent of Direct Effect (InSIDE) assumption, we utilized instrumental variables [IV; Linkage Disequilibrium (LD) *r*^2^ < 0.001, *P* value < 1.0 × 10^−8^, and window size = 10 megabase; refs. 9, 35]. We included 3 sensitivity analyses: heterogeneity test, pleiotropy test, and leave-one-out test (36–39). Based on the sensitivity analysis results, we recommended the appropriate model: (i) no heterogeneity and no pleiotropy: IVW; (ii) heterogeneity but no pleiotropy: IVW-random effect or WM; (iii) pleiotropy: MR-Egger. Our analysis was performed based on the *TwoSampleMR* R package (v0.6.14).

#### Genetic correlation between 12 cancers and 13 metabolic traits

With GWAS summary statistics, we used GNOVA to assess the genome-wide genetic correlations between 12 cancer types and 13 metabolic-related traits (19). GNOVA quantifies the genetic correlation between pairs of traits by estimating their genetic covariance. Additionally, we used LAVA (v0.1.0) to analyze the local genetic correlations between 12 cancer types and 13 obesity-related traits across 2,468 genomic regions (20). In the bivariate analysis (the first step of LAVA), we utilized FDR in the Bonferroni procedure (FDR <0.05) to control the type I error.

#### gSEM for metabolic traits

Using the *GenomicSEM* R package (v0.0.5), we fitted the gSEM (40) to perform confirmatory factor analysis on the 13 exposure factors. gSEM introduces the structural equation modeling framework into genetic analysis, providing a robust methodologic and theoretical foundation for investigating latent variables and their interrelationships. We extracted shared latent factors across groups of exposures and generated GWAS summary statistics for these shared factors. Specifically, we grouped CIHD, stroke, and T2D as a chronic disease exposure set and extracted a shared disease factor (DF). We selected BMI, WHR, and BFP as an obesity-related exposure set and extracted a shared obesity factor (OF). We grouped HDL, LDL, TC, and TG as a lipid metabolism exposure set and extracted a shared lipid factor (LF). Lastly, we grouped HbA1c, glucose, and urate as a metabolic exposure set and extracted a shared metabolic factor (MF). In addition, we conducted genetic correlation and MR analyses between the 4 factors and 12 cancers to further validate and support our findings.

#### PRS constructed by gSEM

To enhance the robustness of PRS, we only retained 121,071 EUR individuals (Supplementary Table S2; Supplementary Fig. S1). Using DBSLMM-auto (22), we constructed PRS for each cancer type and for the 4 shared latent factors. We compared the prediction performances of the null model and integrative model (Eq. A):

$$\begin{array}{c} \left\{ \begin{array}{c} Null\;model:logit\left(P\;=\;1\right)∼{PRS}_{cancer}\\ Integative\;model:logit\left(P\;=\;1\right)∼{PRS}_{cancer}\;+{PRS}_{gSEM} \end{array}\right. \end{array}$$ (A)

where ${PRS}_{cancer}$ indicated the cancer-specific PRS and ${PRS}_{gSEM}$ suggested the PRS constructed by gSEM. We calculated the McFadden R^2^ for each model using the pR2 function in the *pscl* R package (v1.5.9; ref. 41). To compare the improvement between two models, we applied a bootstrap approach with 1,000 iterations. For each iteration, we randomly sampled 50,000 individuals to build the models and calculate the difference in McFadden R^2^ (McFadden R^2^ from the alternative model minus that from the null model). In addition, we used the Nagelkerke R^2^ in the *pscl* R package as a sensitivity analysis (41).

All statistical analyses were performed using R (version 4.3.1) statistical software. A two-sided *P* value was set at < 0.05 to indicate statistical significance. We used the reference panel for 503 EUR individuals from 1000 Genomes Project.

#### Ethics approval and consent to participate

The UK Biobank study was approved by the North West Multi-centre Research Ethics Committee , and all participants provided written informed consent. This study was conducted under UK Biobank application number 144904, using publicly available GWAS summary statistics. We thank the UK Biobank and all participants for their contributions to this research.

## Results

### Prospective cohort analysis

**Figure 2.**
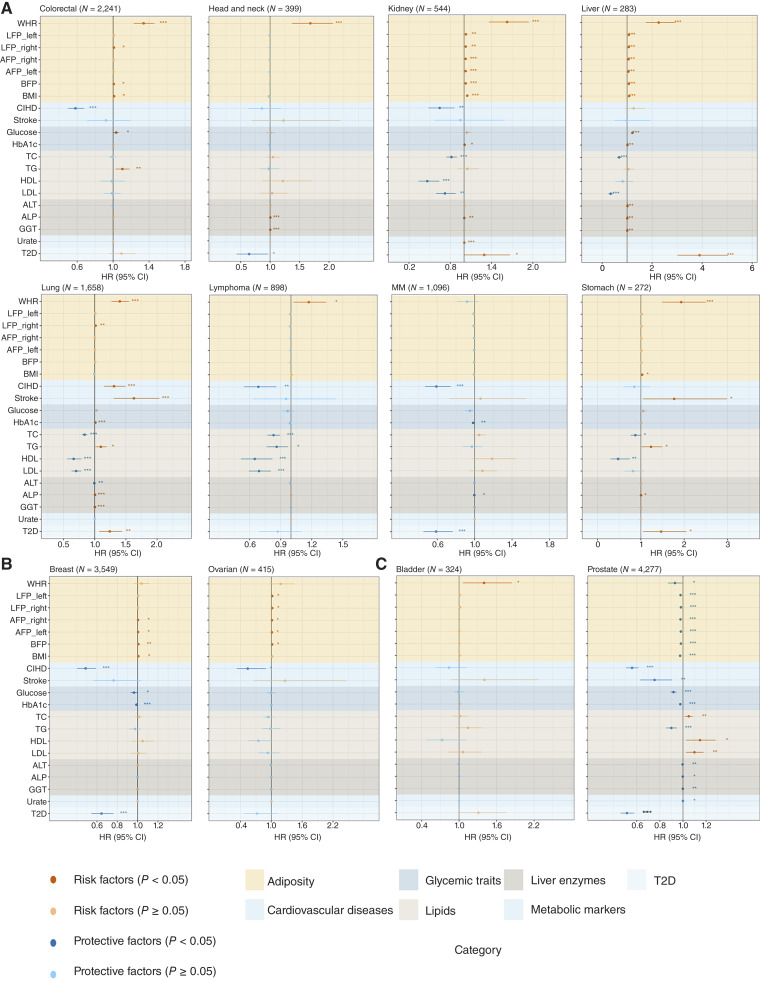
HRs for metabolic trait–cancer pairs. HRs for each metabolic trait–cancer pair were estimated using Cox regression models. For sex-specific cancers, models were adjusted for age, lifescore, and ancestry; for non–sex-specific cancers, models were additionally adjusted for sex. Error bars represent 95% CIs, with the center points indicating HR estimates. The number of cases for each cancer is shown in parentheses in the top-left corner of each plot. *P values: *, <0.05; **, <0.01; ***, <0.001. Different background colors indicate distinct categories of metabolic traits. **A,** Results for non–sex-specific cancers; (**B**) for female-specific cancers; and (**C**) for male-specific cancers.

We estimated the HR and OR between cancer incidences and each metabolic traits with Cox and logistic regressions, respectively (Fig. 2A–C; Supplementary Tables S5 and S6). The association between GGT and liver cancer was the strongest (HR = 1.0056; 95% CI = 1.0051–1.006; *P* = 2.12 × 10^−132^). For the 7 body obesity indices, namely, WHR, LFP_left, LFP_right, AFP_right, AFP_left, BFP, and BMI, we defined them as risk factors to colorectal cancer, kidney cancer, and liver cancer. For example, all the 7 indices are risk factors to kidney cancer and liver cancer. However, all body obesity indices were protective factors for prostate cancer (e.g., BFP: HR = 0.98; 95% CI = 0.977–0.987; *P* = 1.54 × 10^−11^). Among these, WHR was also a risk factor for 7 cancers, including bladder cancer (HR = 1.40; 95% CI = 1.06–1.85; *P* = 0.02).

For the 4 lipid indices, their effects showed heterogeneity among cancers. In lung cancer and stomach cancer, TC, HDL, and LDL were protective factors, whereas TG acted as a risk factor. We observed similar trends in colorectal cancer, kidney cancer, and liver cancer. For example, we defined TG as a risk factor to colorectal cancer, which is consistent with (42). Conversely, in prostate cancer, TC, HDL, and LDL were risk factors (e.g., TC: HR = 1.05; 95% CI = 1.02–1.08; *P* = 0.003), whereas TG served as a protective factor (HR = 0.90; 95% CI = 0.85–0.95; *P* = 8.16 × 10^−5^).

CIHD, stroke, and T2D were identified as risk factors for lung cancer and protective factors for prostate cancer. Notably, CIHD was also a protective factor for colorectal cancer, lymphoma cancer (LPC), kidney cancer, MM, breast cancer, and ovarian cancer. Stroke and T2D were risk factors for stomach cancer. Importantly, T2D exhibited heterogeneous effects across multiple cancers. It was a risk factor for kidney cancer, liver cancer, and lung cancer (e.g., T2D–lung cancer: HR = 1.24; 95% CI = 1.07–1.44; *P* = 4 × 10^−3^) but a protective factor for head and neck cancer, MM, and breast cancer (e.g., T2D-breast cancer: HR = 0.64; 95% CI = 0.55–0.76; *P* = 2.66 × 10^−7^).

The logistic OR analysis was highly consistent with the Cox regression results (Supplementary Table S6; Supplementary Fig. S2A–S2C). All 7 body obesity indices were also risk factors for colorectal cancer, kidney cancer, and liver cancer. WHR remained a risk factor for head and neck cancer, lung cancer, LPC, stomach cancer, and bladder cancer (e.g., bladder cancer: OR = 1.40; 95% CI = 1.06–1.85; *P* = 0.02). Additionally, the 7 obesity indices were protective factors for prostate cancer (e.g., BFP: OR = 0.98; 95% CI = 0.976–0.987; *P* = 2.03 × 10^−11^). Furthermore, we also found the heterogeneous effects of the 4 lipid indicators on multiple cancers. In lung cancer and stomach cancer, TC, HDL, and LDL were protective factors, whereas TG was a risk factor. Conversely, in prostate cancer, TC, HDL, and LDL were risk factors (e.g., TC–prostate cancer: OR = 1.05; 95% CI, 1.02–1.08; *P* = 0.003), whereas TG was a protective factor (OR = 0.90; 95% CI = 0.85–0.95; *P* = 8.31 × 10^−5^). The heterogeneous effects of T2D across multiple cancers were also consistent with those observed in the Cox regression analysis. T2D was a risk factor for kidney cancer, liver cancer, and lung cancer (e.g., lung cancer: OR = 1.24; 95% CI = 1.07–1.44; *P* = 4 × 10^−3^) but a protective factor for head and neck cancer, MM, and breast cancer (e.g., breast cancer: OR = 0.64; 95% CI = 0.54–0.76; *P* = 3.15 × 10^−7^).

### Casual inference

**Figure 3.**
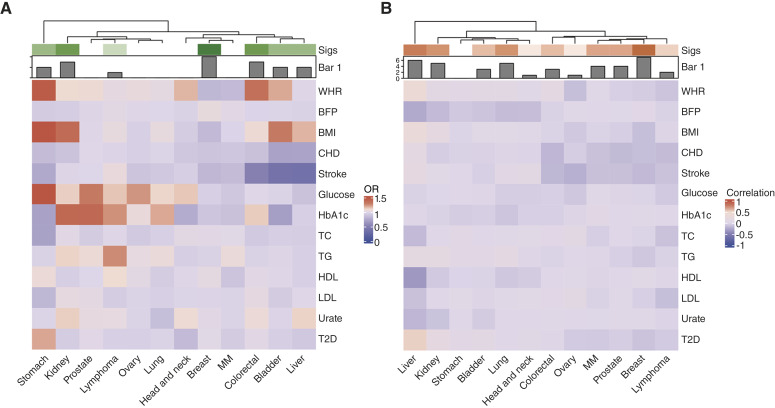
Genetic associations and causal inference between metabolic traits and cancers. **A,** Results of genome-wide genetic correlation analysis using GNOVA. Color intensity from deep blue to deep red indicates increasing genetic correlation from −1 to 1; lighter colors reflect weaker or nonsignificant correlations. Top, bar plot shows the number of significantly associated metabolic traits per cancer before FDR correction. The top clustering dendrogram represents hierarchical clustering of cancers based on their correlation patterns with metabolic traits. **B,** Results of MR causal inference. Color intensity from deep blue to deep red indicates increasing ORs from 0 to 1.5; lighter colors reflect weaker or nonsignificant causal evidence. Top, bar plot shows the number of significantly associated metabolic traits per cancer before FDR correction. The top clustering dendrogram represents hierarchical clustering of cancers based on OR values across metabolic traits. CHD, chronic ischemic heart disease; Sigs, Significant.

The MR causal inference analysis confirmed causal relationships between 17 trait pairs (Fig. 3A). Among these, the association between lung cancer and CIHD was the strongest (OR_MR-Egger_ = 0.72, 95% CI = 0.63–0.82, *P*_MR-WM_ = 1.4 × 10^−6^; MR-Egger intercept = 9.6 × 10^−3^, *P* = 6.2 × 10^−3^). BMI showed significant causal effects on 7 types of cancer. IVW results indicated that BMI was a protective factor for breast cancer (OR_MR-WM_ = 0.83, 95% CI = 0.78–0.89, *P*_MR-WM_ = 2.54 × 10^−7^; MR-Egger intercept = 1.7 × 10^−3^, *P* = 4.5 × 10^−1^), whereas it acted as a risk factor for kidney cancer and liver cancer (e.g., BMI–kidney cancer: OR_MR-IVW_ = 1.39, 95% CI = 1.22–1.59, *P*_MR-IVW_ = 1.21 × 10^−6^; MR-Egger intercept = 3 × 10^−3^, *P* = 2 × 10^−1^). These findings were consistent with previous epidemiologic studies, although some did not reach statistical significance.

CIHD was causally associated with 5 cancer types, and in all cases it showed protective effects (e.g., CIHD–colorectal cancer: OR_MR-WM_ = 0.88, 95% CI = 0.82–0.95, *P*_MR-WM_ = 1.44 × 10^−3^; MR-Egger intercept = 4 × 10^−3^, *P* = 9.1 × 10^−1^). The causal associations between CIHD and colorectal cancer, kidney cancer, and breast cancer had previously been identified in epidemiologic studies, and the direction of effect was consistent. T2D was found to have causal relationships with liver cancer, which also aligned with some findings from epidemiologic studies (T2D–liver cancer: OR_cohort_ = 3.79, 95% CI = 2.93–4.89, *P* = 1.62 × 10^−24^; OR_MR-IVW_ = 1.21, 95% CI = 1.07–1.36, P_MR-IVW_ = 1.54 × 10^−3^; MR-Egger intercept = 6.7 × 10^−3^, *P* = 1.8 × 10^−1^). Among all cancer types, breast cancer had the highest number of causal associations with metabolic traits (4/17, 24%). In addition to BMI and CIHD, both stroke and BFP were identified as important influencing factors (e.g., BFP–breast cancer: OR_corhot_ = 1.01, 95% CI = 1.002–1.012, *P* = 4.43 × 10^−3^; OR_MR-WM_ = 1.05, 95% CI = 1.03–1.07, P_MR-WM_ = 4.27 × 10^−6^; MR-Egger Intercept = 5.3 × 10^−3^, *P* = 6 × 10^−1^). Colorectal cancer followed closely with 3 causal associations (3/17, 18%) for stroke, WHR, and CIHD (e.g., CIHD–colorectal cancer: OR_cohort_ = 0.58, 95% CI = 0.50–0.67, *P* = 6.71 × 10^−13^; OR_MR-WM_ = 0.88, 95% CI = 0.82–0.95, P_MR-WM_ = 1.44 × 10^−3^; MR-Egger intercept = 4 × 10^−3^, *P* = 9.1 × 10^−1^; Supplementary Tables S7–S9).

### Genetic correlation

Using GNOVA and LAVA, we conducted genetic correlation analyses. Through GNOVA analysis with FDR correction using the BH procedure, we identified 41 significantly genetically correlated trait pairs (Fig. 3B). Among these, BMI, T2D, and BFP exhibited the greatest number of significant genetic correlations with various cancers, all showing 6 associations (6/41, 15%). For T2D, liver cancer showed the strongest genetic correlation ($r_{g}\;=\;0.53$; *P* = 4.61 × 10^−22^), which is consistent with findings from cohort analysis (HR = 3.40; *P* = 2.11 × 10^−25^). Among other metabolic traits, the strongest genetic correlation was identified between WHR and liver cancer ($r_{g}\;=\;0.40$; *P* = 8.03 × 10^−4^). TG showed positive genetic correlations with bladder cancer, kidney cancer, and lung cancer (e.g., kidney cancer: $\;r_{g}\;=\;0.17$; *P* = 3 × 10^−4^), whereas it showed a negative genetic correlation with breast cancer (*r*_*g*_ = −0.08; *P* = 1.41 × 10^−3^), which is consistent with cohort findings. Among cancers, breast cancer showed the largest number of genetic correlations with metabolic traits. Among them, stroke exhibited the strongest genetic correlation ($r_{g}\;=\;-0.16$; *P* = 9.78 × 10^−11^).

**Figure 4.**
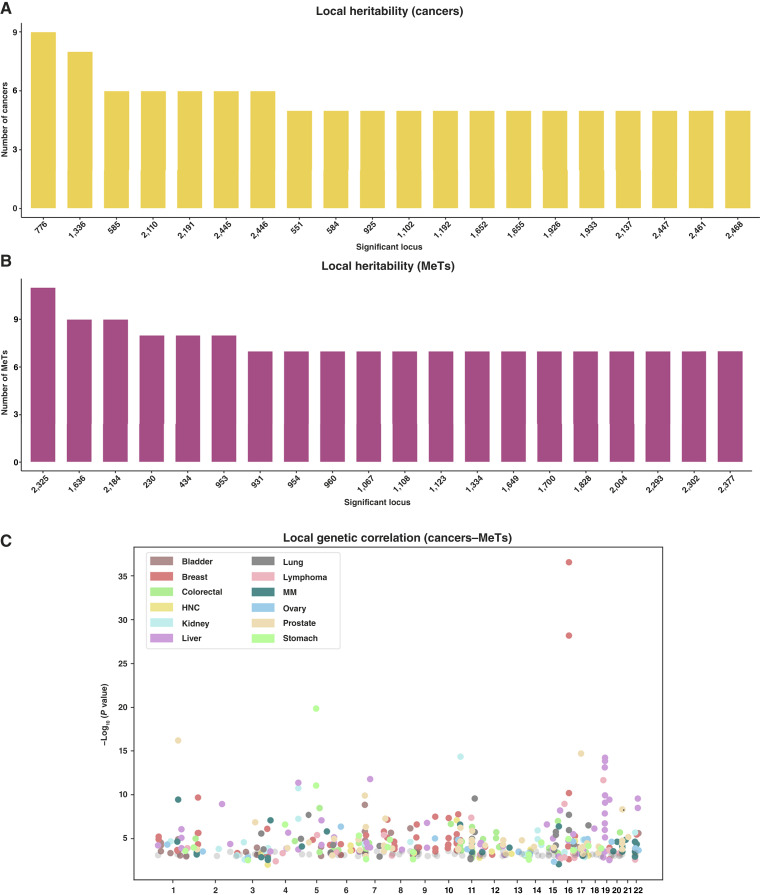
Metabolic trait–cancer associations across multiple genomic regions. **A,** We selected genomic regions showing significant heritability for each cancer at *P* < 0.05/2,468 and displayed the top 20 regions along with the number of cancers showing significant heritability at each locus. **B,** Similarly, we identified genomic regions with significant heritability for each metabolic trait at *P* < 0.05/2,468 and presented the top 20 regions and the number of metabolic traits showing significant heritability at each locus. **C,** Manhattan plots illustrating LAVA results for cancer and metabolic traits. Points in different colors represent different cancers. HNC, head and neck cancer. Mets, metabolic traits.

Furthermore, we investigated local genetic correlations between different cancers and metabolic traits across 2,468 genomic regions. We first performed univariate analyses for each genomic region of each trait to identify those with significant local heritability (Fig. 4A and B). Then, we defined 405 genomic regions showing bivariate genetic association signals across 100 trait pairs. Among these, breast cancer and BMI exhibited significant genetic correlations across the largest number of genomic regions (49 regions), followed by breast cancer and T2D (48 regions), prostate cancer and BMI (41 regions), and prostate cancer and T2D (40 regions). Among the metabolic traits, BMI (240/733, 32.7%) and T2D (241/733, 32.9%) exhibited the highest number of bivariate association signals, followed by CIHD (121/733, 16.5%). The fewest association signals were observed for TG and WHR, with only 3 each. Among cancer traits, breast cancer (132/733, 18%), prostate cancer (121/733, 16.5%), and colorectal cancer (66/733, 9%) showed the highest number of bivariate association signals. In contrast, lymphoma (34/733, 5%) and head and neck cancer (15/733, 2%) had the lowest number of association signals (Fig. 4C; Supplementary Tables S10 and S11).

Notably, region 1655 (chr11: 68,887,345–69,713,996) and region 1652 (chr11: 64,594,823–66,782,661) showed the most significant local genetic correlation signals, spanning a total of 17 trait pairs (Fig. 4C). Among them, region 776 (chr5: 1,206,610–2,106,885) and region 2325 (chr19: 45,040,933–45,893,307) contained the highest number of univariate genetic signals for cancer and metabolic traits, respectively (Fig. 4A and B).

### gSEM

**Figure 5.**
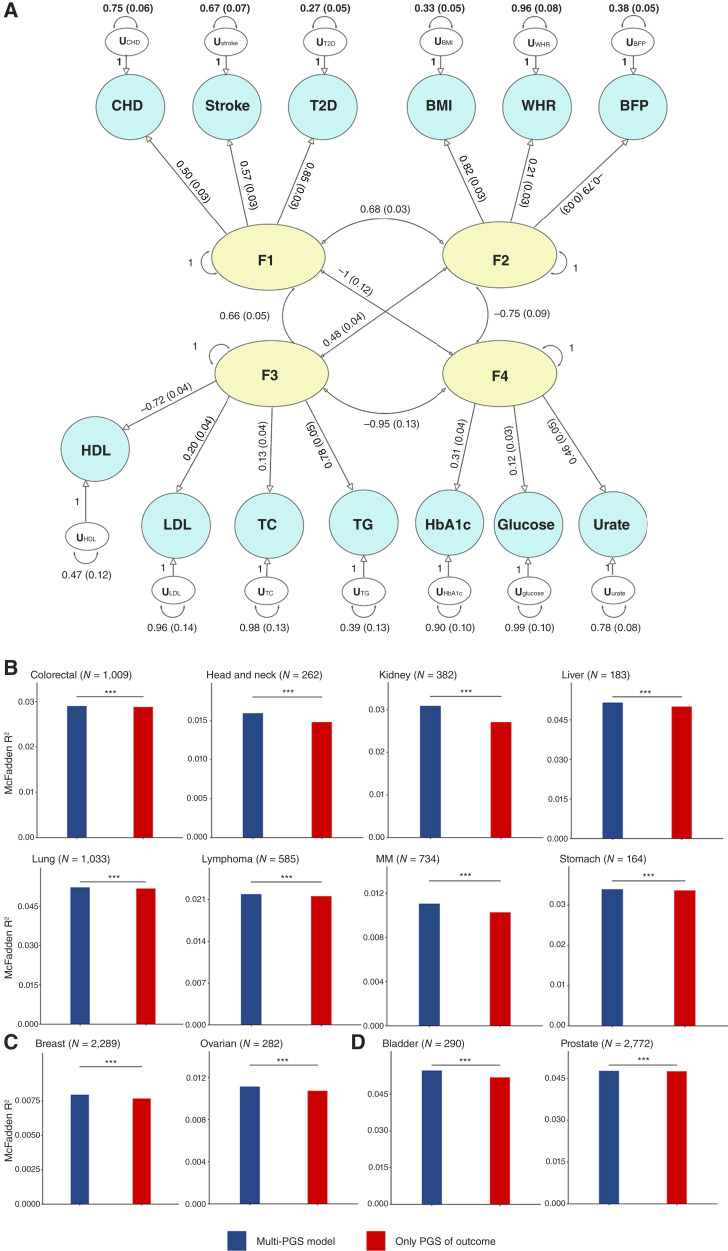
gSEM construction and cancer risk prediction evaluation. **A,** Path diagram of the gSEM. Yellow ellipses represent latent variables (i.e., extracted common factors), blue circles denote observed variables (i.e., metabolic traits), and white ellipses represent residual terms. Unidirectional arrows from latent variables to observed variables indicate standardized regression coefficients (factor loadings). Bidirectional arrows between latent variables represent pairwise correlations. **B,** Bar plot comparing the predictive performance of the null model (red) and the alternative model (blue) for non–sex-specific cancers. **C,** Bar plot comparing the predictive performance for female-specific cancers. **D,** Bar plot comparing the predictive performance for male-specific cancers. Stars above error bars indicate significance levels from bootstrap tests: ***, *P* < 0.001. CHD, chronic ischemic heart disease.

We conducted a gSEM analysis for the 13 metabolic traits (Fig. 5A). Among the latent factors identified, the DF showed the strongest contribution to T2D (β = 0.85), followed by stroke (β = 0.57) and CIHD (β = 0.50). The OF had the highest loading on BMI (β = 0.82), followed by BFP (β = −0.79) and WHR (β = 0.21). The LF exhibited the strongest influence on TG (β = 0.78) and HDL (β = −0.72), with relatively weaker contributions to LDL (β = 0.20) and TC (β = 0.13). Lastly, the MF showed the highest loading on urate (β = 0.46), followed by HbA1c (β = 0.31) and glucose (β = 0.12). Notably, there were significant genetic correlations among the 4 common latent factors. The DF and MF showed a strong negative genetic correlation ($r_{g}\;=\;-1$). DF also correlated moderately with the OF ($r_{g}\;=\;0.68$) and the LF ($r_{g}\;=\;0.66$). The OF was strongly negatively correlated with the MF ($r_{g}\;=\;-0.75$), positively correlated with the LF ($r_{g}\;=\;0.48$), and moderately correlated with the DF. Additionally, the LF and MF exhibited a strong negative genetic correlation ($r_{g}\;=\;-0.95$). Importantly, the MF showed high negative genetic correlations with all 3 other latent factors (Supplementary Tables S12 and S13).

### PRS

We constructed 2 PRSs for 12 types of cancer (Fig. 5B–D). Specifically, we constructed the integrative PRS model based on only three common latent factors (the MF was excluded because of its high genetic correlations with the other three factors). We used McFadden R^2^ as the primary evaluation metric for 2 models. Bootstrap validation results demonstrated that across all predicted cancer traits, the integrative PRS models consistently outperformed the null models, highlighting the added predictive value of incorporating multiple metabolic-related traits and latent factors. For example, the McFadden R^2^ of kidney cancer for the 2 models were 0.0272 and 0.0310 (*P* < 1 × 10^−8^), respectively, indicating a 13.95% improvement in model fit. We conducted a sensitivity analysis using the Nagelkerke R^2^ and yielded the same conclusions as those of the above results (Supplementary Tables S14 and S15; Supplementary Fig. S3A–S3C).

## Discussion

Here, integrating the large-scale prospective cohort and genomic analyses, we profiled the onco-metabolic nexus and used it to improve prediction performance. The nexus was constructed with both shared genetic architecture with genetic correlation and causal relationships using MR and cohort analyses. Based on the potential causal pathway, we introduced gSEM to integrate the GWAS summary statistics of metabolic traits, constructed the integrative nexus, and defined the improvement of integrative PRS models across 12 types of cancer.

In previous epidemiologic studies, lipids such as TG, HDL, LDL, and TC were commonly reported to be associated with cancer risk, though their effect directions are not entirely consistent (43–46). In line with (47), we defined TG as a risk factor for colorectal cancer, whereas HDL, LDL, and TC had no significant associations with colorectal cancer. Besides colorectal cancer, we observed that TG often exerted opposite effects compared with other lipids, particularly in lung cancer, stomach cancer, and prostate cancer. Notably, all 4 lipid indicators were protective against LPC, a finding that is consistent with previous reports (48, 49). In kidney cancer and liver cancer, TG tended to increase cancer risk, although the results were not statistically significant; LDL and TC were protective factors, and HDL was also protective for kidney cancer. These findings suggest that different lipid components may influence cancer risk through distinct mechanisms and pathways. Moreover, the heterogeneous impact of these lipid markers across cancer types—such as HDL being a protective factor for most cancers but a risk factor for prostate cancer—reveals the complexity of lipid metabolism in carcinogenesis. Further research is required to uncover the underlying biological mechanisms and to understand why the same metabolic trait exhibits opposing effects in different cancers. Unlike lipid traits, obesity-related indicators demonstrated more homogeneous associations with cancer risk. Traits including WHR, LFP_left, LFP_right, AFP_left, AFP_right, BFP, and BMI were consistently associated with increased risks of colorectal cancer, kidney cancer, and liver cancer, highlighting the pivotal role of obesity in cancer progression and the importance of weight management for cancer prevention. Interestingly, all obesity-related indicators were inversely associated with prostate cancer, which aligns with the findings of Neal and colleagues (50–52). This suggests that obesity may have a protective effect on prostate cancer, potentially contributing to male prostate health. Future studies with larger sample sizes are needed to confirm this observation and explore the potential mechanisms involved. Among chronic disease indicators, CIHD, T2D, and stroke exhibited protective effects in several cancers, such as CIHD–kidney cancer, T2D–head and neck cancer, and stroke–prostate cancer, possibly due to lifestyle modifications, medication use, or systemic changes in patients with chronic conditions (53, 54). However, all three conditions were linked to increased risk of lung cancer, likely related to behavioral factors such as smoking and alcohol consumption (55).

Besides perspective analysis, we performed MR and genetic correlation using GWAS summary statistics to mutually reinforce each other, providing strong evidence for future investigations. MR provided further causal support for multiple metabolic–cancer trait pairs, corroborating earlier findings (56, 57), suggesting that their roles may differ before and after diagnosis (58). Genome-wide genetic association analyses identified 41 significant trait pairs. Among these, several pairs were consistent with previous reports, such as BMI–breast cancer (59, 60). Interestingly, liver cancer and T2D have been identified as a trait pair with significant correlation in epidemiologic studies, genetic correlation analyses, and MR analyses, exhibiting consistent effect directions across all three methods.

To better integrate the onco-metabolic nexus into clinical applications, we explored the feasibility of incorporating these metabolic traits into predictive models. In the “gSEM + PRS” framework, we constructed polygenic scores (PGS) based on common latent factors among metabolic traits and integrated them into prediction. Our results indicated that compared with null models, most integrative PRS models enhanced prediction performance. Like psycho-metabolic nexus, the finding indicates that integrating PRS derived from metabolic traits can improve cancer risk prediction (17).

With regard to the strong negative correlation observed between the OF and MF, we propose the following potential explanations. On the one hand, the current GWAS data for HbA1c, glucose, and urate are limited by relatively small sample sizes and few SNPs, which may lead to estimation bias. On the other hand, the OF and MF may exhibit genetic reverse pleiotropy, meaning that some variants increase the MF while others reducing the OF and vice versa. We look forward to the release of larger-scale metabolism-related GWAS in the future to improve statistical power.

The integrated PRS is constructed by extracting shared factors from multiple variables to explore the association between these shared genetic factors and the outcome. We believe that this approach offers several advantages. First, constructing the PRS using shared factors from multiple variables effectively avoids the problem of multicollinearity that arises when multiple correlated variables are directly included in a statistical model. Second, the integrated PRS can be interpreted as the shared genetic component of several variables. Investigating the association between this shared genetic component and the outcome can lay the groundwork for subsequent exploration of specific biological mechanisms, for example, how certain genes may influence several metabolic markers and thereby contribute to cancer development. We believe that this approach can advance the implementation of precision medicine.

The main limitations of the integrated PRS at this stage include the accuracy of variable grouping, which must be clinically and biologically meaningful. In addition, as different genetic structures correspond to different model assumptions, training an optimal PRS model for the integrated PRS remains a challenge.

Our study has several limitations. First, genomic analyses were primarily conducted in EUR individuals, limiting the generalizability of our findings to other ethnic groups. As is well known, large-scale GWASs are available only for EUR individuals, which can provide enough power to our study. Second, some traits were excluded from cohort and genomic analyses. It might be due to insufficient low incident rates or traits not eligible for heritability estimation. Finally, we did not have independent validation cohorts to externally verify our findings.

### Conclusion

Integrating cohort analysis and genome-wide pleiotropy analysis, our study identified multiple metabolic traits associated with specific cancers. By combining gSEM and DBSLMM methods, we innovatively proposed a new strategy for integrating metabolic traits into predictive models. Our results demonstrate that incorporating PGS derived from metabolic traits can consistently enhance cancer risk prediction performance, emphasizing their value in early diagnosis and personalized treatment. The interplay between cancer and metabolic traits underscores the need to consider both domains in future research and public health strategies.

## Supplementary Material

Supplementary Table S1 Description of UKBB cohort data for cohort analysis.

Supplementary Table S2 Description of UKBB cohort data for PGS analysis.

Supplementary Table S3 Summary of 31 GWAS without UKBB individuals summary statistics.

Supplementary Table S4 Heritability of 31 GWAS without UKBB individuals summary statistics.

Supplementary Table S5 Cox regression HRs of cohort analysis.

Supplementary Table S6 Logistic regression ORs of cohort analysis.

Supplementary Table S7 MR IVW, Weighted Median, and MR Egger Results for Onco-Metabolic Traits.

Supplementary Table S8 Heterogeneity Test Results for Onco-Metabolic Traits in Mendelian Randomization.

Supplementary Table S9 Pleiotropy Test Results for Onco-Metabolic Traits in Mendelian Randomization.

Supplementary Table S10 Global genetic correlations between onco-metabolic traits.

Supplementary Table S11 Regional genetic correlations between onco-metabolic traits through LAVA.

Supplementary Table S12 GenomicSEM Results for Metabolic Traits.

Supplementary Table S13 Model fit indices of the genomic structural equation model.

Supplementary Table S14 Comparison of Model Predictive Performance with and without Metabolic Factors Using McFadden's R².

Supplementary Table S15 Comparison of Model Predictive Performance with and without Metabolic Factors Using Nagelkerke's R².

Supplementary Figure S1 Flowchart of Sample Screening and Selection Process.

Supplementary Figure S2 Odds ratios for metabolic trait–cancer pairs.

Supplementary Figure S3 Sensitivity analysis of cancer risk prediction evaluation.

## Data Availability

The data used in this study were obtained from multiple publicly available genetic and epidemiologic resources, including UKB (approval application number: 144904, http://ukbiobank.ac.uk/register-apply/); FinnGen r12 (https://r12.finngen.fi/); GIANT (https://giant-consortium.web.broadinstitute.org/index.php/GIANT_consortium_data_files); Global Lipids Genetics Consortium (https://csg.sph.umich.edu/willer/public/glgc-lipids2021/); the UK Household Longitudinal Study (https://www.understandingsociety.ac.uk/); and CKDGen Consortium (https://ckdgen.imbi.uni-freiburg.de/). This study complies with all ethical guidelines and data use agreements of each database. All raw data generated in the study are available upon reasonable request to the corresponding author.
