# Supplementary material for: Profiling the Onco-metabolic Nexus and Improving Cancer Risk Prediction Performance: A Large-scale Cohort and Genome-Wide Pleiotropic Analysis
Source: Cancer Res Commun. 2026 May 8;6(5):1071–82. doi: 10.1158/2767-9764.CRC-26-0099 (PMC13153864; doi:10.1158/2767-9764.CRC-26-0099)
Supplement: Supplementary Figure S1 — Flowchart of Sample Screening and Selection Process. [file crc-26-0099_supplementary_figure_s1_suppsf1.docx]

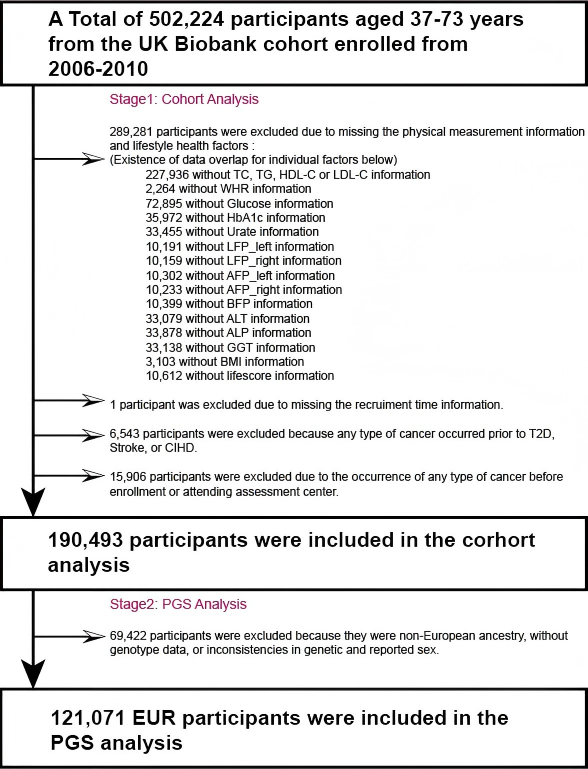


**Supplementary Figure S1 Flowchart of Sample Screening and Selection Process.**

We first excluded individuals with missing relevant information from the initial 502,224 UK Biobank samples, leaving 190,493 samples for cohort analysis. In the PGS analysis, we further removed non-European ancestry individuals and retained only those of European ancestry, resulting in a final sample size of 121,071.
