# Supplementary material for: Profiling the Onco-metabolic Nexus and Improving Cancer Risk Prediction Performance: A Large-scale Cohort and Genome-Wide Pleiotropic Analysis
Source: Cancer Res Commun. 2026 May 8;6(5):1071–82. doi: 10.1158/2767-9764.CRC-26-0099 (PMC13153864; doi:10.1158/2767-9764.CRC-26-0099)
Supplement: Supplementary Figure S2 — Odds ratios for metabolic trait–cancer pairs. [file crc-26-0099_supplementary_figure_s2_suppsf2.docx]

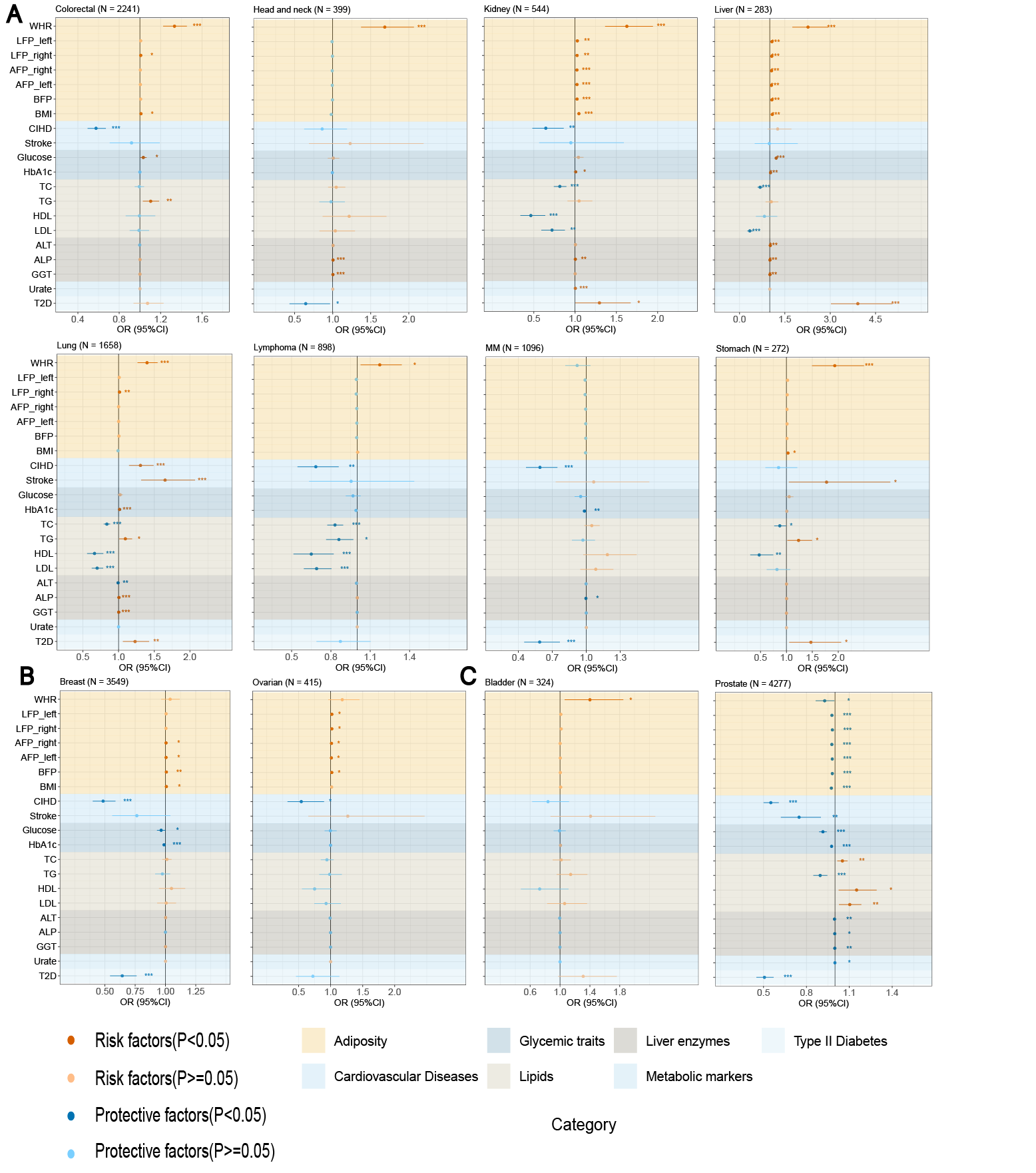


**Supplementary Figure S2 Odds ratios for metabolic trait–cancer pairs.**

Odds ratios (ORs) for each metabolic trait–cancer pair were estimated using Logistic regression models. For sex-specific cancers, models were adjusted for age, lifescore, and ancestry; for non-sex-specific cancers, models were additionally adjusted for sex. Error bars represent 95% confidence intervals (CIs), with the center points indicating HR estimates. The number of cases for each cancer is shown in parentheses in the top-left corner of each plot. *P values: * < 0.05, ** < 0.01, *** < 0.001. Different background colors indicate distinct categories of metabolic traits. **Panel A** shows results for non-sex-specific cancers; **Panel B** for female-specific cancers; and **Panel C** for male-specific cancers.
