# Supplementary material for: Profiling the Onco-metabolic Nexus and Improving Cancer Risk Prediction Performance: A Large-scale Cohort and Genome-Wide Pleiotropic Analysis
Source: Cancer Res Commun. 2026 May 8;6(5):1071–82. doi: 10.1158/2767-9764.CRC-26-0099 (PMC13153864; doi:10.1158/2767-9764.CRC-26-0099)
Supplement: Supplementary Figure S3 — Sensitivity analysis of cancer risk prediction evaluation. [file crc-26-0099_supplementary_figure_s3_suppsf3.docx]

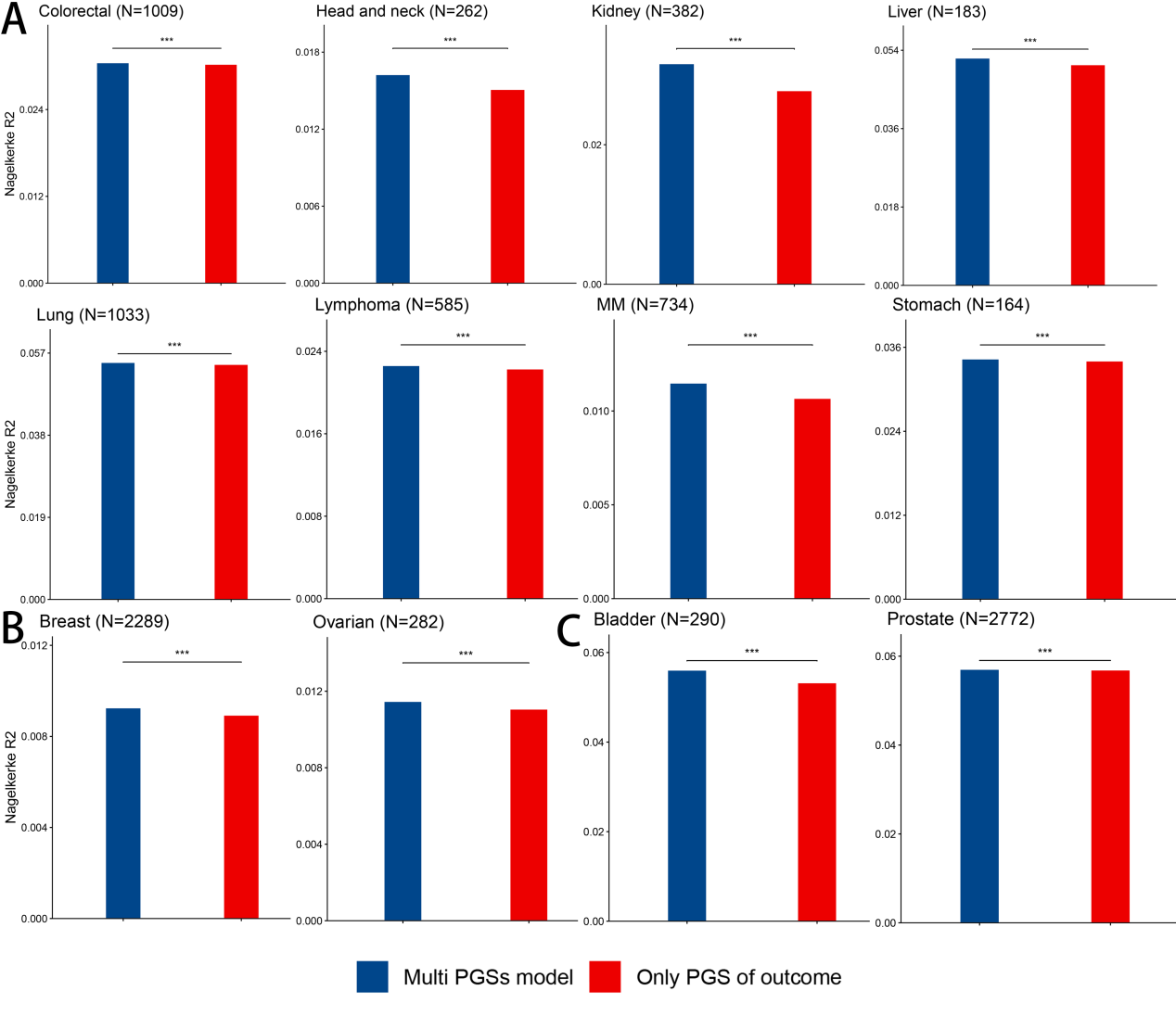


**Supplementary Figure S3 Sensitivity analysis of cancer risk prediction evaluation.**

Bar plot comparing the predictive performance of the null model (red) and the alternative model (blue). The null model included only cancer-specific PRS, while the alternative model incorporated both cancer-specific and metabolic factor–derived polygenic scores (PGSs). Stars above error bars indicate significance levels from bootstrap tests: *P < 0.05, **P < 0.01, ***P < 0.001. **Panel A** shows results for non-sex-specific cancers; **Panel B** for female-specific cancers; and **Panel C** for male-specific cancers.
